# Association Between Hyperacusis and Tinnitus: Analysis of Prevalence and Impact on Daily Functioning

**DOI:** 10.3390/audiolres16050126

**Published:** 2026-08-26

**Authors:** Snežana Babac, Aleksandar Perić, Vladan Milutinović, Jasmina Stojanović, Ivana Ilić-Savić, Emilija Živković Marinkov, Svetlana Valjarevic, Luka Čučić, Zorana Radin

**Affiliations:** 1 Clinic of Otorhinolaryngology, Clinical and Hospital Centre “Zvezdara,” 11120 Belgrade, Serbia; vladan.milutinovic@stom-f.bg.ac.rs (V.M.); lukacucic17@gmail.com (L.Č.); zorana.radin@gmail.com (Z.R.); 2 Faculty of Special Education and Rehabilitation, University of Belgrade, 11000 Belgrade, Serbia; ivanailicsavic@fasper.bg.ac.rs; 3 Faculty of Medicine of the Military Medical Academy, University of Defense, 11000 Belgrade, Serbia; aleksandarperic1971@gmail.com; 4 Faculty of Dental Medicine, University of Belgrade, 11000 Belgrade, Serbia; 5 Department of Otorhinolaryngology, Faculty of Medical Sciences, University of Kragujevac, 34000 Kragujevac, Serbia; orl@ukck.rs; 6 Clinic Otorhinolaryngology, Clinical Center Niš, 18000 Niš, Serbia; emilija.zivkovic.marinkov@medfak.ni.ac.rs; 7 Clinical Hospital Center Zemun, Faculty of Medicine, University of Belgrade, 11080 Zemun, Serbia; svetlana.valjarevic@med.bg.ac.rs

**Keywords:** tinnitus, hyperacusis, prevalence, quality of life, THI

## Abstract

**Background**: Hyperacusis is a common, but often underrecognized comorbidity in patients with chronic subjective tinnitus. Reduced sound tolerance is associated with a greater tinnitus-related burden, impaired communication, reduced social participation, and poorer daily functioning. However, published data regarding its prevalence and clinical impact remain limited and inconsistent. **Objectives**: To determine the prevalence of hyperacusis in patients with chronic subjective tinnitus and to assess its association with tinnitus-related handicap, uncomfortable loudness levels, and degree of hearing loss and daily functioning. **Methods**: This cross-sectional study included 194 patients aged 21–85 years with chronic subjective tinnitus. Hyperacusis was assessed using a structured clinical interview that included questions on decreased sound tolerance and sound-induced ear pain and uncomfortable loudness level measurements (ULLs). Patients were categorized into two groups: tinnitus with hyperacusis (T + H; *n* = 32) and tinnitus without hyperacusis (T; *n* = 162). The impact of tinnitus on daily functioning was evaluated using a standardized and clinically validated questionnaire—the Tinnitus Handicap Inventory (THI). Data were analyzed using appropriate parametric and nonparametric statistical tests. **Results**: Hyperacusis was identified in 32 of 194 patients with chronic tinnitus (16.5%). Patients with tinnitus and hyperacusis (T + H) had significantly higher THI scores than those with isolated tinnitus, indicating greater tinnitus-related handicap (76.3 vs. 41.9; *p* < 0.001). Patients with hyperacusis also demonstrated significantly lower ULL values than those without hyperacusis (*p* < 0.001). **Conclusions**: Although the reported prevalence of hyperacusis among patients with tinnitus varies across the literature, our findings indicate that hyperacusis is a common comorbidity associated with significantly greater tinnitus-related handicap. Routine assessment of hyperacusis, including ULL measurements, should be incorporated into the clinical evaluation of patients with tinnitus to facilitate comprehensive diagnosis and optimize management. These findings highlight the importance of systematic assessment of hyperacusis in patients with tinnitus in routine audiological practice.

## 1. Introduction

Tinnitus and hyperacusis are common auditory conditions characterized by altered sound perception and reduced sound tolerance, respectively, both of which may negatively affect daily functioning, especially when they occur together. Hyperacusis is characterized by reduced sound tolerance and increased sensitivity to everyday environmental sounds of low-to-moderate intensity, which most individuals do not perceive as loud [1]. Tinnitus represents the perception of sound in the absence of an external acoustic stimulus [2,3]. The coexistence of these conditions has been associated with increased tinnitus-related handicap, impaired communication, reduced social participation, and poorer quality of life. Although the association between tinnitus and hyperacusis has been previously described, studies have demonstrated that co-occurring hyperacusis is associated with greater tinnitus-related handicap and reduced sound tolerance [1,4]. Therefore, identifying hyperacusis in patients presenting with chronic tinnitus may provide clinically relevant information for patient assessment and management. The present study evaluated hyperacusis, tinnitus-related handicap, uncomfortable loudness levels, and degree of hearing loss within the same clinical cohort, with particular emphasis on the clinical value of assessing patients with tinnitus for co-occurring hyperacusis.

The aim of this study was to determine the prevalence of hyperacusis in patients with chronic subjective tinnitus and to evaluate its association with tinnitus-related handicap, uncomfortable loudness levels, and degree of hearing loss.

## 2. Materials and Methods

This cross-sectional study included 194 consecutive patients aged 21–85 years with chronic subjective non-pulsatile tinnitus who were prospectively enrolled and examined from January 2024 to July 2024 at the Department of Audiovestibulology, Clinic of Otorhinolaryngology, Clinical and Hospital Centre, Zvezdara. All participants were referred to the audiology outpatient clinic because chronic subjective tinnitus was their primary complaint. Patients with acute-onset tinnitus, tinnitus associated with sudden sensorineural hearing loss, Ménière’s disease, otosclerosis, acoustic trauma, pulsatile tinnitus, vestibular disorders, or other identifiable otologic diseases that could account for tinnitus were excluded from study. Chronic subjective tinnitus was defined as the perception of tinnitus lasting for 6 months or longer. Patients were classified into two groups based on the presence of hyperacusis: tinnitus with hyperacusis (T + H, *n* = 32) and tinnitus without hyperacusis (T, *n* = 162). As there are currently no universally accepted diagnostic criteria for hyperacusis, the diagnostic approach used in this study was based on a structured clinical interview combined with ULL measurements, consistent with previously published literature [4,5,6,7]. ULL values of ≤90 dB HL at two or more frequencies were used as a supportive criterion for hyperacusis (250–8000 Hz) [4,5,6,7]. ULLs were measured at octave frequencies between 250 and 8000 Hz using pure-tone stimuli. A validated hyperacusis-specific questionnaire was not administered because it was not part of the routine clinical assessment protocol during the study period. All patients underwent otorhinolaryngological examination, pure-tone audiometry, and ULL testing. Tinnitus-related handicap was assessed using the validated Tinnitus Handicap Inventory (THI), which provides a total score ranging from 0 to 100, with higher scores indicating greater tinnitus-related handicap [8]. All patients completed the THI questionnaire independently before audiological assessment. The degree of hearing loss was determined based on better-hearing ear using pure-tone average (PTA; 500–4000 Hz) according to the World Health Organization (WHO) classification [9]. Data were processed by descriptive and inferential statistics using IBM SPSS Statistics version 22.0. Parametric variables were analyzed using Student’s *t*-test or Mann–Whitney *U* test depending on data distribution, while categorical variables were compared using the chi-squared test. Correlations were assessed using Spearman’ s correlation coefficient. A *p* value < 0.05 was considered statistically significant.

The study was approved by the Institutional Ethics Committee, and written informed consent was obtained from all patients prior to enrollment. Patients did not receive financial compensation for their participation.

## 3. Results

Out of the total sample (194 patients with chronic subjective tinnitus), tinnitus with associated hyperacusis (T + H) was confirmed in 32 patients (16.5%), while isolated tinnitus (T) was present in 162 patients (83.5%). The mean age was 51.2 ± 13.2 years in the T + H group and 54.1 ± 15.3 years in the T group, with no statistically significant difference between groups (t = −1.24, *p* = 0.22). Likewise, the distribution of age categories was comparable between the T + H and T group (χ^2^ = 3.62, df = 2; *p* > 0.05). Nevertheless, patients aged 21–40 years represented a larger proportion of the T + H group (31.3%) than of the tinnitus-only group (23.5%). The overall sample included 58.2% males, with no significant gender differences between the T + H and T groups (χ^2^ = 0.048; df = 1; *p* > 0.05). The distribution of subjects by degree of hearing loss is shown in Table 1. A statistically significant difference was observed between the T + H and T groups (χ^2^ = 8.06; df = 3; *p* < 0.05). In the T + H group, a higher proportion of patients had a normal hearing threshold (37.5%), whereas severe/very severe hearing loss predominated in the T group (35.2%).

In the T + H group, both tinnitus and hyperacusis were bilateral in all patients. In the T group, tinnitus was unilateral in 91 patients (56.2%) and bilateral in 71 patients (43.8%). This difference was not statistically significant (χ^2^ = 2.47, df = 1, *p* > 0.05). Among patients unilateral tinnitus, 49 had left-sided and 42 had right-sided tinnitus, with no significant difference between sides (χ^2^ = 0.54, df = 1, *p* > 0.05).

Patients in the T + H group had significantly higher mean THI scores (76.3 ± 12.1; very severe handicap) than patients in the T group (41.9 ± 12.3; mild-to-moderate handicap) (Table 2). The difference was highly significant (Mann–Whitney U test, *p* < 0.01). The total THI scores ranged from 54 to 100 in the T + H group, corresponding to moderate-to-catastrophic handicap, and from 16 to 68 in the T group, corresponding to slight-to-severe handicap. In the T + H group of patients, lower mean ULL values were recorded, averaging (from 125–8000 Hz) 74.8 compared to 91.6 in the T group (Table 2). Statistical analysis (independent sample *t*-test) showed that the differences in ULL values between the groups were highly statistically significant (*p* < 0.01).

**Figure 1:**
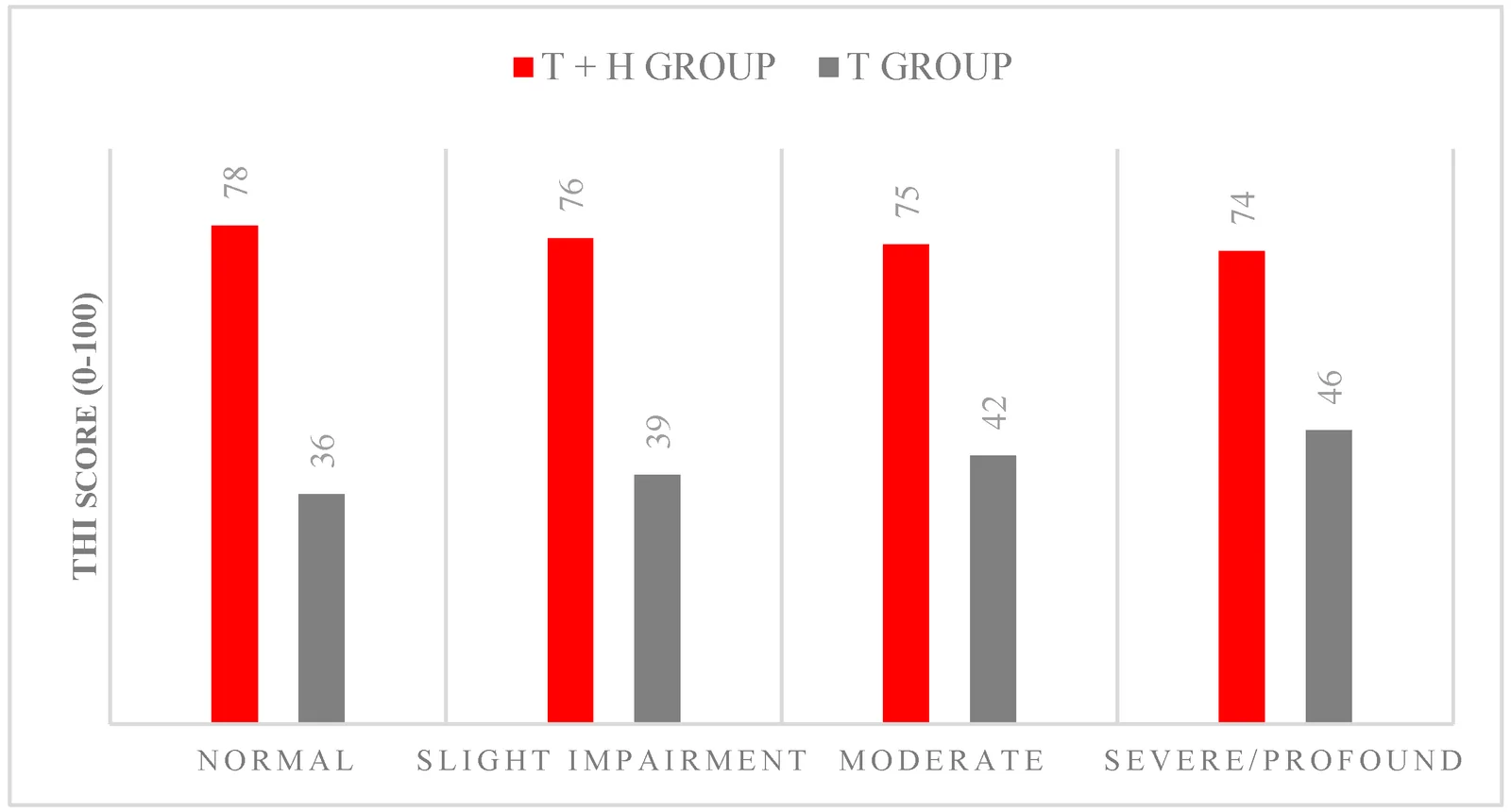
THI in relation to the degree of hearing loss and participant group.

A two-way ANOVA with two independent factors, group (T + H and T) and degree of hearing loss (normal, mild, moderate, severe/profound), revealed statistically significant differences in THI scores. THI scores increased with increasing degree of hearing loss in the tinnitus-only (T) group of patients (Figure 1). The main effect of group was significant: F (1, 186) = 1458.22, *p* < 0.001, indicating that patients with hyperacusis have significantly higher THI values compared to patients without hyperacusis, regardless of the degree of hearing loss. The main effect of degree of hearing loss was also significant: F (3, 186) = 9.60, *p* < 0.001. A significant interaction between group and degree of hearing loss was also observed—F (3, 186) = 10.13, *p* < 0.001—indicating that the association between degree of hearing loss and THI scores differed according to hyperacusis status. In patients with hyperacusis, high THI scores were observed even among those with normal hearing thresholds, whereas in patients without hyperacusis, THI scores increased with increasing severity of hearing loss.

In order to examine the association between the subjective severity of tinnitus and ULLs, a correlation analysis was performed between THI scores and ULL values. The results showed a statistically significant negative correlation between these two parameters (Spearman’s correlation coefficient ρ = −0.7; *p* < 0.01), indicating that higher THI scores were associated with lower ULL values. The THI subscale with the highest mean score in the T + H group of patients was the functional subscale (32.8), followed by the emotional subscale (27.1) and then the catastrophic subscale (16.4). In the tinnitus-only group, the highest score was also observed on the functional subscale (22.3), followed by the emotional subscale (13.4) and the catastrophic subscale (5.5) (Table 2). To compare THI subscale scores between groups, the Mann–Whitney U test was used. All subscale scores in the tinnitus-only group were lower compared to the T + H group. Statistically significant differences were found across all three subscales (*p* < 0.01 for all). In the T group, patients with bilateral tinnitus had significantly higher mean THI scores than those with unilateral tinnitus (45 ± 13 vs. 39 ± 11, respectively; t = −3.03; *p* < 0.01). Analysis of THI scores by sex showed that women had higher mean THI scores than men in both groups. In the T + H group, the difference was not statistically significant (78.3 vs. 74.3; t = 0.94 *p* = 0.35), whereas in the T group, women had significantly higher THI scores than men (44.4 vs. 39.4; t = 2.6; *p* = 0.01).

## 4. Discussion

Hyperacusis, a disorder of decreased tolerance to sounds of moderate intensity, when coexisting with tinnitus, may intensify discomfort, anxiety, helplessness, sleep disturbances, fatigue, negative emotions, and concentration difficulties [10]. Additionally, hyperacusis is often associated with hearing loss [11]. Previous studies have reported a wide range of hyperacusis prevalence among patients with tinnitus, reflecting differences in diagnostic criteria and study populations. In our study, hyperacusis was present in 16.5% of patients, which is comparable to the prevalence reported by Fabijanska (15.2%) and Guimarães (18.4%) [12,13]. However, higher prevalence rates have also been described in other cohorts, ranging from 30% to 55%, emphasizing methodological differences across studies [14,15,16,17]. Their findings suggest that although hyperacusis is relatively common in individuals with tinnitus, it does not necessarily correlate with a higher degree of subjectively perceived distress caused by tinnitus itself. In contrast, in our study, a significant correlation between hyperacusis and tinnitus with higher THI scores was confirmed.

In our study, males predominated in the overall sample, consistent with previous epidemiological studies reporting a higher prevalence of tinnitus among men, largely attributed to greater lifetime occupational noise exposure (e.g., military, police, construction, industry) [18,19,20]. Sex distribution did not differ significantly between the T + H and T groups, suggesting that sex is unlikely to be a dominant factor in the development of hyperacusis as a comorbidity of tinnitus. Similarly, only a limited number of previous studies have reported a male predominance among patients with combined tinnitus and hyperacusis [13,21]. The mean age of patients in both groups was similar—51.2 years in the group of patients with hyperacusis and tinnitus and 54.1 years in the group with isolated tinnitus—as was the distribution of age groups (*p* > 0.05). However, a higher proportion of younger patients was observed in the group with combined hyperacusis and tinnitus (31.3%) compared to patients with isolated tinnitus (23.5%). This difference in age distribution suggests a potential predisposition of younger patients to develop combined symptoms of tinnitus and hyperacusis [22]. However, this observation should be interpreted with caution because the difference was not statistically significant. The higher proportion of younger adults in the T + H group may be due to a potentially higher sensitivity of younger patients to auditory stimuli and greater psychophysiological load, which can increase the likelihood of developing hyperacusis with tinnitus [17,23]. An alternative explanation may be earlier help-seeking among younger patients with co-occurring tinnitus and hyperacusis, as the coexistence of these symptoms has been associated with greater tinnitus-related handicap [17,24]. Recent studies have also shown that patients with concomitant tinnitus and hyperacusis are generally younger, experience higher levels of mental and general distress associated with tinnitus, and have a higher prevalence of painful disorders compared to patients with isolated tinnitus [24]. However, the poorer subjective hearing reported in that study was not observed in our cohort.

The distribution of hearing-loss severity differed significantly between the T + H and T groups. Notably, 37.5% of patients with concomitant tinnitus and hyperacusis had normal audiometric thresholds. This finding indicates that hyperacusis is not restricted to patients with hearing loss and may occur in individuals with normal pure-tone thresholds. This finding is consistent with previous reports indicating that hyperacusis may occur despite normal audiometric thresholds, suggesting that mechanisms other than peripheral hearing loss may contribute to hyperacusis [25,26,27]. In contrast, among patients with isolated tinnitus, moderate-to-severe/profound hearing loss was more frequently observed, suggesting an association between tinnitus and peripheral auditory deficits. Clinically, these findings emphasize that evaluation of patients with tinnitus should include assessment of ULLs, in addition to medical history-taking and pure-tone audiometry. As expected, patients in the T + H group demonstrated significantly lower mean ULL values than those in the tinnitus-only group (74.8 vs.91.6 dB HL). This finding is consistent with the reduced sound tolerance characteristic of hyperacusis and with previous reports of lower ULLs in affected patients [5].

In our study, patients with both tinnitus and hyperacusis had a significantly higher total THI score (76.3—very severe handicap) compared to patients with isolated tinnitus (41.9—mild-to-moderate handicap). Studies by other authors also indicate that the presence of hyperacusis further increases the experience of discomfort and social and emotional dysfunction in patients with tinnitus [17,24,28]. According to Andersson and colleagues, patients with tinnitus and concomitant hyperacusis exhibit higher levels of stress and psychological distress compared to patients with tinnitus alone [29]. Hyperacusis may enhance attention directed toward internal auditory sensations, making tinnitus perceived as more intense and disturbing [23]. All THI subscales, functional, emotional, and catastrophic, were significantly higher in patients with combined hyperacusis and tinnitus. In both groups, the functional subscale showed the highest scores, indicating that these symptoms primarily affect the patient’s ability to perform daily activities. Our findings support the concept that the coexistence of hyperacusis and tinnitus represents a more complex auditory and psycho-emotional condition requiring a broader diagnostic and therapeutic approach [24,30]. Our study demonstrated significantly lower mean uncomfortable loudness levels (ULLs) in patients with hyperacusis than in those without hyperacusis (*p* < 0.01), consistent with previous studies identifying reduced ULLs as an objective characteristic of hyperacusis [31,32]. Both hearing-loss severity and the presence of hyperacusis were significantly associated with THI scores. Across all degrees of hearing loss, patients with combined tinnitus and hyperacusis had markedly higher THI scores (73–78) than patients with isolated tinnitus (40–45). However, in patients with hyperacusis, THI scores remained high even in the presence of normal hearing thresholds, indicating that the presence of hyperacusis was more strongly associated with higher THI scores than the degree of hearing loss itself. In patients with isolated tinnitus, THI scores increased proportionally with the severity of hearing loss. These findings are consistent with studies suggesting that psychoacoustic characteristics of sound are not the only factors affecting tinnitus perception and quality of life, but that the presence of associated symptoms such as hyperacusis also plays a significant role [33].

Tinnitus laterality was also associated with tinnitus-related handicap in our cohort. In the T group, patients with bilateral tinnitus had significantly higher THI scores than those with unilateral tinnitus (45 ± 13 vs. 39 ± 11, *p* < 0.01). This finding is consistent with a previous study reporting significantly higher THI scores in patients with bilateral compared with unilateral tinnitus [34,35]. However, findings in the literature are not uniform. In contrast to our results, Song et al. reported significantly higher THI scores in patients with unilateral tinnitus than in those with bilateral tinnitus [36]. These discrepancies may reflect differences in study populations, hearing status, and other clinical characteristics. Notably, in the T + H group, both tinnitus and hyperacusis were bilateral in all patients. This finding is consistent with previous reports indicating that hyperacusis commonly presents as a bilateral phenomenon [16,37]. Taken together, these findings suggest that tinnitus laterality may represent an additional factor associated with tinnitus-related handicap and should be considered when interpreting THI scores. In our cohort, among patients with unilateral tinnitus, left-sided tinnitus was slightly more frequent than right-sided (49 vs. 42 patients). However, this difference was not statistically significant (*p* > 0.05). This finding is consistent with the reported tendency toward left-sided predominance, which has been attributed to greater susceptibility of the left cochlea to noise damage and asymmetry in the medial olivocochlear efferent system [35,38], although no significant lateral predominance was observed in our sample. We found a strong negative correlation between THI scores and mean ULL values (*p* < 0.01), indicating that higher subjective severity of tinnitus is associated with lower sound tolerance thresholds. This association further emphasizes the clinical relevance of ULL assessment in patients with severe tinnitus. Although ULL measurements are widely used in the clinical assessment of hyperacusis, the procedure should be performed cautiously, because high-intensity sound stimulation may provoke discomfort or distress in susceptible patients. Therefore, testing should always be stopped immediately when the patient indicates intolerance, and patient safety should remain the primary consideration. In addition, although validated hyperacusis-information questionnaires may provide valuable information regarding the subjective impact of hyperacusis, our diagnostic approach was based on a structured clinical interview combined with ULL assessment, reflecting the routine clinical protocol used in our institution. We acknowledge that validated hyperacusis-specific questionnaires, such as the Hyperacusis Questionnaire (HQ), may provide additional information regarding the subjective impact of hyperacusis and should be considered in future studies [39].

Women demonstrated higher THI scores than men in both study groups, reaching statistical significance only in patients with isolated tinnitus. Our findings are consistent with previous studies suggesting that women tend to report greater tinnitus-related emotional distress, sleep disturbances, and anxiety related to tinnitus [3,40]. Additionally, women more often seek medical care and provide more detailed descriptions of their complaints, which may result in higher subjective ratings of symptom severity [40,41].

This study has several limitations. First, it was conducted at a single center and had a cross-sectional design, which limits the generalizability of the findings and precludes conclusions regarding causal relationships. Second, the subgroup of patients with concomitant tinnitus and hyperacusis was relatively small (*n* = 32), which may limit the statistical power of subgroup analyses. Hyperacusis was identified using a structured clinical interview combined with ULL measurements without the use of a validated hyperacusis-specific questionnaire. Although tinnitus laterality was recorded and analyzed, tinnitus duration beyond the predefined inclusion criterion (>6 months) was not further evaluated in relation to THI scores. In addition, psychological comorbidities, medication use, and occupational noise exposure were not systematically assessed in relation to THI scores and may have influenced the observed associations. Future prospective multicenter studies with larger samples should include validated hyperacusis questionnaires, systematically assess these potential confounding factors, and incorporate them into multivariable analyses.

## 5. Conclusions

The prevalence of hyperacusis among patients with tinnitus in our study was 16.5%. Although hearing-loss distribution differed between groups, hyperacusis was also observed in patients with normal audiometric thresholds, indicating that hearing loss is not a prerequisite for its occurrence. In contrast, among patients with isolated tinnitus, higher THI scores were associated with greater hearing-loss severity. This observation highlights the need for a more comprehensive diagnostic approach in patients with hyperacusis and normal audiometric results, including routine assessment of sound tolerance (ULLs) in addition to case history and pure-tone audiometry. Patients with concomitant tinnitus and hyperacusis experienced substantially greater tinnitus-related handicap than those with isolated tinnitus, as reflected by significantly higher total THI scores and higher scores across the functional, emotional, and catastrophic subscales. Furthermore, ULL values below 90 dB HL at two or more frequencies may serve as a useful clinical indicator of hyperacusis. Otorhinolaryngologists and audiologists should routinely assess sound tolerance in patients presenting with tinnitus to improve recognition of concomitant hyperacusis and enhance clinical management.

## Figures and Tables

**Table 1:** Prevalence of hearing loss by group.

Degree of Hearing Loss	T + H		T		Total
	N	%	N	%	
Normal threshold	12	37.5%	29	17.9%	41 (21.1%)
Mild	8	25.0%	41	25.3%	49 (25.3%)
Moderate	7	21.9%	35	21.6%	42 (21.6%)
Severe and very severe	5	15.6%	57	35.2%	62 (32.0%)
Total	32	16.5%	162	83.5%	194 (100%)

**Table 2:** Comparison of THI total and subscale scores and mean ULL values between groups.

Variable	T + H Group (*n* = 32), Mean ± SD	T Group (*n* = 162), Mean ± SD	*p*
Total THI	76.3 ± 12.1	41.9 ± 12.3	<0.01
Functional THI	32.8 ± 13.2	22.6 ± 12.7	<0.01
Emotional THI	27.1 ± 8.5	13.6 ± 5.8	<0.01
Catastrophic THI	16.4 ± 14.7	5.7 ± 2.5	<0.01
Mean ULL (dB HL)	74.8 ± 8.2	91.6 ± 7.5	<0.01

## Data Availability

The original contributions presented in this study are included in the article. Further inquiries can be directed to the corresponding author.

## Abbreviations

The following abbreviations are used in this manuscript:

THI	Tinnitus Handicap Inventory
ULL	uncomfortable loudness level
PTA	pure-one average
